# Draft Genome Sequence of an *Oceanobacillus* sp. Strain Isolated from Soil in a Burial Crypt

**DOI:** 10.1128/genomeA.00701-16

**Published:** 2016-07-28

**Authors:** Ilargi Martínez-Ballesteros, Ylenia Arizaga, Joseba Bikandi, Javier Garaizar, Giulia Ganau, Bianca Paglietti, Manuela Murgia, Massimo Deligios, Salvatore Rubino

**Affiliations:** a Department of Immunology, Microbiology and Parasitology, University of the Basque Country UPV/EHU, Vitoria-Gasteiz, Spain; b Department of Biomedical Sciences, University of Sassari, Sassari, Italy; c Department of Infection and Immunity, King Faisal Specialist Hospital and Research Centre, Riyadh, Saudi Arabia

## Abstract

We present the draft genome of an *Oceanobacillus* sp. strain isolated from spores found in soil samples from a burial crypt of the Cathedral of Sant'Antonio Abate in Castelsardo, Italy. The data obtained indicated the closest relation of the strain with *Oceanobacillus caeni*.

## GENOME ANNOUNCEMENT

It is widely known that microorganisms can inhabit deep ocean water in spite of the extreme conditions. *Oceanobacillus* is one of the bacteria living under these conditions, and even though bacteria of this genus have been isolated from different environments (1–4), they are frequently found in marine-related habitats (5–11). The *Oceanobacillus* genus consists of Gram-positive, endospore-forming, and moderately halophilic bacteria. The *Oceanobacillus* strain of this study was isolated in 2011 from the soil of a burial crypt in the Cathedral of Sant'Antonio Abate in Castelsardo (Sardinia, Italy). The crypt contained buried bodies, some of which naturally mummified, that were interred between the year 1600 and at least 1830, and they have remained sealed until the opening of the crypt in 2011.

Soil samples were collected during the excavation of the crypt by bioarchaeologists equipped with N-95 masks, gloves, and Tyvek suits. Isolation of spore-forming bacteria was performed from the soil samples according to a selective protocol for *Bacillus* species (12). One of the colonies isolated was identified by 16S rRNA gene sequencing (13) as *Oceanobacillus caeni*. 16S rRNA gene was analyzed using the SINA alignment Service (14) and BLAST (http://blast.ncbi.nlm.nih.gov/) with the nonredundant database to find the best homology. Genome sequencing of the strain was also performed after DNA extraction from an overnight growth at 37°C on Luria broth (LB) using the NucleoSpin tissue kit (Macherey Nagel, Germany), according to the manufacturer’s protocol. Sequencing was performed using Illumina MiSeq platform (Illumina, San Diego, CA), obtaining 3,866,671 reads with an average length of 209 bp. The data generated were *de novo* assembled with Edena (15), with k-mer of 140 nucleotides in length, resulting in 150 contigs. These contigs were manually analyzed, and our own scripts were generated to search matching sequences of the reads at both ends of the contigs. The amount of contigs was reduced to 46, with a genome total length of 3,731,305 bp and an average of G+C content of 35.75%. The largest contig was 843,196 bp in length, and more than half of the contigs were >1,000 bp. Annotation of the genome was then performed using the Rapid Annotations using Subsystems Technology server (RAST) (16, 17). A total of 3,661 coding sequences (CDSs) and 131 tRNAs were predicted. As determined by the RAST server, 438 subsystems were in the genome.

The coverage of 13 reference 16S rRNA genes from *Oceanobacillus* species obtained from the Ribosomal Database Project (18) with the reads obtained in this project was searched. The best coverage was obtained for *Oceanobacillus caeni*, but the coverage was not complete. An alignment-free approach based on octanucleotide content of genomes was then used to identify the most closely related organism. The GScompare platform was used for this purpose (http://gscompare.ehu.eus/) (J. Bikandi, unpublished data), and again, the results indicated *Oceanobacillus caeni* to be the most closely related genome.

### Nucleotide sequence accession numbers.

This whole-genome shotgun project has been deposited in DDBJ/ENA/GenBank under the accession no. LYCS00000000. The version described in this paper is version LYCS01000000.
